# When is too small, too small? Evaluating the trade-offs of smaller dairy cows in pasture-based systems

**DOI:** 10.3168/jdsc.2025-0935

**Published:** 2025-12-18

**Authors:** D.P. Berry, R.D. Evans

**Affiliations:** 1 Teagasc, Animal & Grassland Research and Innovation Centre, Moorepark, Fermoy, Co. Cork, P61 P302, Ireland; 2 Irish Cattle Breeding Federation, Ballincollig, Co. Cork, P31 D452, Ireland

## Abstract

Interest in dairy cow size as a determinant of overall production efficiency is intensifying globally across different production systems, particularly in pasture-based systems where energy intake is constrained. Smaller cows are theoretically more efficient, as a lower proportion of their energy intake is required for maintenance. However, concerns persist that reducing genetic merit for cow live weight may compromise productivity, both in terms of milk yield and progeny carcass weight. A key concern is that output could decrease rapidly if live weight drops below a critical threshold. The objective of the present study was to quantify the association between genetic merit for live weight and phenotypic performance for milk production and progeny carcass weight in Irish pasture-based Holstein-Friesian cows. Following all edits, phenotypic 305-d milk yield, fat yield, and protein yield were available on 831,307 lactations from 583,361 cows calving in 2023 and 2024, whereas live weight records were available for 27,600 cows, with carcass weight data available for 49,906 of their progeny. Cows were stratified into 20 groups based on live weight PTA, and associations with milk production traits and progeny carcass weight were quantified using linear mixed models. Phenotypic cow live weight increased almost linearly with genetic merit for live weight across all parities. Milk yield and milk solids yield also increased with live weight PTA, at rates of ~1.1%, 1.3%, and 1.3% per 10-kg increase in live weight PTA for parities 1, 2, and 3, respectively. Milk protein concentration did not change with cow live weight PTA but milk fat concentration reduced as cows got genetically heavier. Progeny carcass weight increased linearly with dam live weight PTA. In all, within the range of cow sizes typical of pasture-based systems, genetically lighter Holstein-Friesian cows produced marginally less milk and had lighter progeny, but no evidence of a threshold below which productivity declined rapidly was detected. These results suggest that moderate genetic reductions in cow live weight may improve herd efficiency without materially compromising milk solids or progeny carcass output. Therefore, given that the increase in output associated with genetically heavier cows was modest compared with the potential increased maintenance costs of heavier cows, the economic advantage of selecting for heavier cows in pasture-based systems may be limited.

## Highlights

• Milk solids lactation yield increases with genetic merit for live weight.
• Milk solids per kilogram live weight declined with increased genetic merit for live weight.
• Progeny carcass weight increased with dam live weight genetic merit.
• Overall, genetically heavier had modest output gains but higher maintenance costs.

## Graphical Abstract

The objective of this cross-sectional data analysis study was to explore how the size of dairy cows, reflected by their genetic merit for live weight, relates to milk production and the carcass weight of their offspring. As anticipated, cows genetically expected to weigh more were indeed, on average, heavier. The relationship between cow genetic merit for live weight and milk output was mostly linear, meaning that, as cows got genetically lighter, their milk output reduced steadily but without any sudden drop. Importantly, though, genetically lighter cows were more efficient producers in that they produced more milk solids per kilogram of live weight. The progeny from genetically heavier cows also had slightly heavier carcasses, reflecting the genetic link between cow live weight and offspring growth. In all, the additional output from genetically heavier cows was small compared with their expected extra feed and maintenance needs. Overall, the findings suggest that, in grass-based dairy systems, breeding for moderately smaller cows could improve efficiency while maintaining production.

**Figure:**
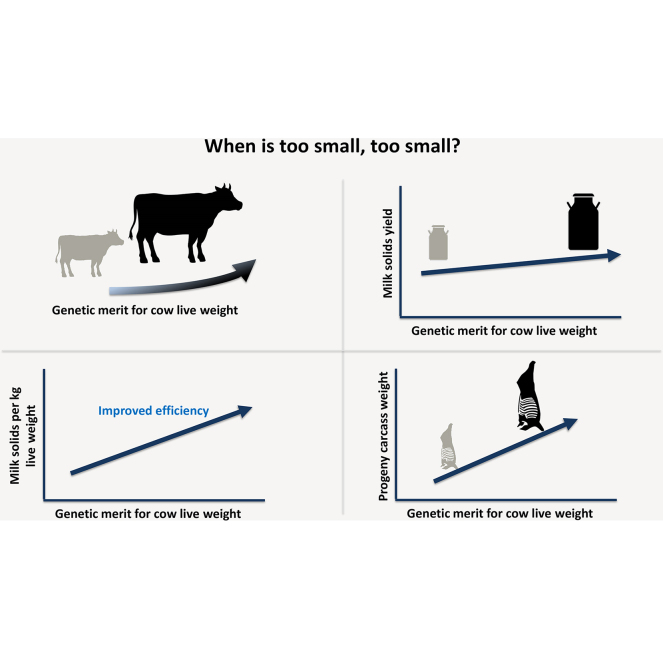

Interest in dairy cow size as a factor influencing overall efficiency is intensifying globally, particularly within pastoral dairy cow production systems (Berry, 2026). Pasture-based dairy cows are generally lighter than cows managed in confinement production systems. All else being equal, smaller cows are generally expected to be more efficient, as a lower proportion of their ingested energy is allocated to body maintenance. However, genetic selection for lighter cows, or indeed maintaining the current cow size, has its skeptics. Legitimate concerns exist that reducing cow size may adversely affect overall output. Output incorporates both milk production and progeny carcass weight, with the latter assuming growing importance due to the expanding role of beef-on-dairy production (Berry, 2021). Of real concern is whether a lower critical threshold of cow live weight exists, below which performance may rapidly deteriorate.

The objective of the present study was therefore to explore the mean lactation milk production of pasture-based Holstein-Friesian dairy cows stratified on genetic merit for live weight; also of interest was the mean carcass weight of their progeny. In addition to average performance, the study also examined the variability in performance among cows of differing genetic merit for live weight. The findings from this study will provide valuable insights for critically assessing the potential impact of negative genetic selection for cow live weight on overall performance.

Lactation (i.e., 305-d) phenotypic milk yield, fat yield, and protein yield were available on 855,700 Irish cows calving in the years 2023 and 2024 inclusive; all cows were recorded to be at least 87.5% Holstein-Friesian. Milk solids yield was calculated as the sum of the fat and protein yields. Only lactations where the cow calved within 6 mo of the median age at calving for that parity were retained; 1,028,753 lactations from 697,917 cows remained. Cow parity was classified as 1, 2, 3, 4, 5, 6, 7 to 10, and >10.

Cow live weight records collected in the 2023 and 2024 calendar years were also available for a selection of these cows; live weight was recorded using electronic scales operated directly by the farmer or through external service providers. Additionally, carcass weight phenotypes were also available for all animals slaughtered in Irish abattoirs up to October 15, 2025; carcass weight is measured in kilograms, on average 1 h after slaughter, following the removal of the head, hide, legs, thoracic and abdominal organs, and internal fat. Of interest in the present study was the progeny of cows slaughtered as either heifers, steers, or young bulls.

Contemporary group for the cow performance traits was defined as herd-year-season of calving using an algorithm used in the Irish national genetic evaluations. Of the cows in the dataset, 98% calved in the first 4 mo of the year. Within each herd, the algorithm clusters cows calving within a 10-d period. If the initial cluster contains fewer than 10 animals, it is merged with an adjacent contemporary group to form a larger group. This process is repeated until the contemporary group comprises at least 10 cows, provided that the interval between the earliest and latest calving dates within the herd does not exceed 30 d. Data from 114,556 cows were discarded because they were in small contemporary groups.

Following all filters, production data from 831,307 lactations from 583,361 cows remained. Of these, phenotypic live weight data were available on 27,810 lactations from 27,600 cows. The live weight measurement nearest to 150 DIM per lactation was retained, with records <10 d postcalving or >305 DIM being excluded. Stage of lactation (percent of data) was subsequently categorized into approximately 30-d intervals from 10 to 30 d (3.0%), 31 to 60 d (4.0%), 61 to 90 d (4.7%), 91 to 120 d (7.4%), 121 to 150 d (16.2%), 151 to 180 d (15.3%), 181 to 210 d (10.1%), 211 to 240 d (11.0%), 241 to 270 d (13.4%), and 271 to 305 d (14.9%). Mean live weight across the lactation stages varied from 546.7 kg in the first stage to 538.8 kg in the second stage (i.e., 31 to 60 DIM) increasing consistently thereafter to 600.3 kg in the last stage of lactation (i.e., 271 to 305 d).

Only carcass weight data from animals born in 2023 and slaughtered in 2024 and 2025 were retained; carcass weight records were available on 100,619 progeny of the cows in the filtered dataset. Contemporary group for carcass weight was defined using the same algorithm already described based on herd-sex-date of slaughter; only contemporary groups with at least 5 records were retained. Carcass weight data were available from 49,906 progeny of the 583,361 cows.

Predicted transmitting ability for live weight, milk production, and carcass weight were obtained from the December 2022 national genetic evaluation. The genetic evaluations for cow live weight and progeny carcass weight are both a multibreed, multitrait genetic evaluation including both dairy and beef cattle together; a repeatability model is used for cow live weight where heterosis, parity, DIM, and age within parity are included as fixed effects along with contemporary group. The genetic evaluation for milk, fat, and protein yield is based on separate random regression test-day model with each parity (i.e., 1, 2, 3+) included as separate traits. The eventual PTA is the weighted average of the 3 parities: 0.41 parity 1, 0.33 parity 2, and 0.26 parity 3+.

Lactations in the final dataset were stratified into 20 groups based on cow PTA for live weight. The PTA for live weight was standardized and a variable derived (to be later used as a covariate in the statistical model) representing 20 equally spaced points across −2 to +2 SD to impose equal distance between strata. The inclusion of this term as a covariate in the statistical model facilitated modeling of linear differences in performance across the different live weight PTA strata.

The associations between stratum of PTA for cow live weight and a selection of performance traits (i.e., milk production, cow live weight, and progeny carcass weight) was quantified using a series of linear mixed model in AsReml (Gilmour et al., 2009). In all models, both contemporary group and cow were included as random effects. Heterogeneous variance by stratum of live weight PTA were modeled for all traits. Furthermore, in all models fitted, the standardized live weight PTA covariate was always included as a fixed effect. Both live weight PTA stratum and parity were included as fixed effects in the model when the dependent variable was a milk production trait. When the dependent variable was live weight, then stage of lactation was also included as a fixed effect in an interaction with parity. Fixed effects included in the model for progeny carcass weight (along with cow live weight PTA stratum and the standardized live weight PTA covariate) were cow parity and age at slaughter in a 2-way interaction with animal sex, where sex was either a heifer, steer, or young bull; the PTA for carcass weight of the sire of the progeny was also included.

The raw mean phenotypic cow live weight in the sample population used in the present study was 560.7 kg with an SD of 73.8 kg. Cows were, on average, weighed at 183 DIM. Lactations of parity 1, 2, 3, and greater than 3 represented 23%, 21%, 17%, and 48% of the dataset, respectively. The mean (SD) 305-d milk yield, fat yield, and protein yield was 6,428.85 (1,185.09) kg, 274.1 (47.2) kg, and 230.6 (40.1) kg, respectively. The raw (SD) mean carcass weight of the 38,325 heifers, 58,179 steers, and 12,571 young bulls was 266.7 (39.3) kg, 312.8 (45.5) kg, and 305.9 (48.6) kg, respectively. The pasture-based Holstein-Friesian cows in the present study would be considered light relative to Holstein (and Holstein-Friesian) cows in the United States (640.50 kg from cows of multiple lactations, of which 45% were primiparous; Toghiani et al., 2024), the Netherlands (667 kg for second-parity cows; van Breukelen et al., 2025), and Denmark (683.8 kg for cows of multiple parities; Lassen et al., 2023). The cows in the present study are, however, slightly heavier than the reported live weight of mid-lactation multiparous New Zealand Holstein-Friesian dairy cows of ~490 kg (Roche et al., 2006); New Zealand, like Ireland, operates a pastoral dairy production system.

As expected, phenotypic cow live weight increased almost consistently with each stratum increase in cow PTA for live weight; the associations for the first 3 parities are shown in Figure 1, with the trends persisting for higher parity cows. Although the association differed by cow parity (*P* < 0.001), the general shape of the association between live weight PTA and phenotypic live weight was relatively consistent across parities. This points to the fact that genetic differences in live weight behave predictably across parities. Second-parity cows were, on average, 10% heavier than their first-parity contemporaries, whereas third-parity cows were, on average, 17% heavier than their first-parity contemporaries. Older parity cows were 22% to 28% heavier than first-parity cows. In a cross-sectional analysis of 10,757 Irish cows with phenotypic live weight records, none of which overlapped with the present study, Ring et al. (2021) reported a linear regression coefficient of 1.00 when regressing phenotypic live weight (adjusted for fixed and random effects) on estimated breeding value for live weight. When PTA live weight in the present study was actually fitted as a continuous variable in the model (without PTA stratum) the regression coefficient was 2.22 (SE = 0.03) which is close to the expectation of 2; when in a 2-way interaction with parity, the regression coefficients were 1.68 (SE = 0.07), 1.91 (SE = 0.069), 2.20 (SE = 0.078), 2.37 (SE = 0.078), 2.54 (SE = 0.075), 2.63 (SE = 0.0829), 2.34 (SE = 0.065), and 2.40 (SE = 0.30) for parity 1, 2, 3, 4, 5, 6, 7 to 10, and greater than 10, respectively. This increase in regression coefficient suggests that genetic potential for cow live weight is more fully expressed in older cows; no interaction existed between the PTA covariate and PTA stratum indicating that the interaction observed with parity number was more a function of maturity than cow size per se. The regression coefficients do, however, clearly confirm the robustness of the national genetic evaluation for cow live weight and its ability to accurate predict the phenotypic differences of groups of cows. The residual variance of live weight did not vary much by PTA strata with the residual SD being between 22.1 kg and 24.1 kg across the 20 live weight PTA strata. Hence, although cows genetically expected to be lighter were indeed, on average, lighter, there was as much variability among them as among cows that were genetically expected to be heavier. Some of this can be attributed to the fact that the genetic merit for individual cows is an estimate and their true genetic merit may deviate from their expectation (but the mean estimate of genetic merit of a group should equal the true genetic merit of that group when estimated using best linear unbiased prediction), but also that the heritability of live weight in cows is only 0.50 (Maskal et al., 2024), implying that nongenetic factors over and above those accounted for in the statistical model can affect actual phenotypic live weight.

**Figure 1:**
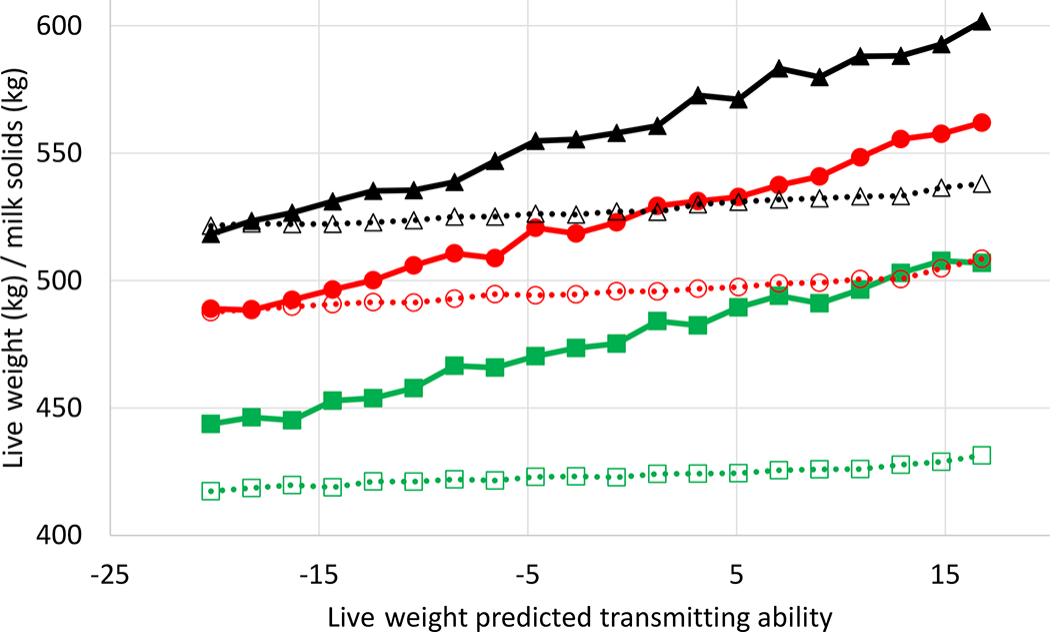
Mean live weight (filled-in legends with continuous lines) and 305-d milk solids yield (open legends with broken lines) for parity 1 (squares), parity 2 (circles), and parity 3 (triangles) cows across different strata of PTA for live weight; SEM were all ≤0.48 kg.

The predicted marginal mean 305-d milk yield by live weight PTA stratum is in Figure 1; the number of lactation records per live weight PTA stratum for the milk production traits varied from 38,280 to 46,317. Milk yield increased at a rate of 1.1%, 1.3%, and 1.3% in parity 1, 2, and 3 cows, respectively, per 10-kg increase in live weight PTA, up to the last 2 PTA strata, where the percentage increase in 305-d milk yield increased more rapidly (Figure 2). The mean difference in milk yield between the extreme live weight PTA strata was 320 kg, 453 kg, and 388 kg for parity 1, 2, and 3 cows, respectively; the mean difference in estimated breeding value (i.e., twice the PTA) for milk yield was 456 kg, implying that the phenotypic difference observed was mostly due simply to a difference in genetic merit of the cows for milk yield. Nonetheless, when the sample population was adjusted for genetic differences in milk yield, live weight PTA remained associated (*P* < 0.001) with phenotypic milk yield. Interestingly, despite the large study size, the association between 305-d milk yield and PTA for milk yield did not differ (*P* = 0.07) by live weight PTA stratum. Therefore, this means that the response to selection for milk production in pastoral production systems does not depend on the genetic merit for live weight of the cow, or indeed the actual phenotypic live weight of the cow. In other words, genetic selection for milk can occur without indirectly favoring larger or smaller cows in pasture systems. Whether this conclusion holds in confinement cows, which tend to be both larger and produce more milk, has not yet been explored. Unlike pasture-based systems, confinement dairy production systems typically provide unrestricted feed supply, enabling larger cows to more readily meet their energy needs for both higher milk output and the greater maintenance costs. The residual SD for milk yield varied from 453 kg to 473 kg across the different live weight PTA strata, except for the 2 heaviest strata, where the residual SD was 509 and 543 kg, respectively. The greater variability among genetically heavier cows may reflect increased heterogeneity in their physiological capacity or feed conversion efficiency but could also be a reflection of these cows being exposed to greater heterogeneity in herd management.

**Figure 2:**
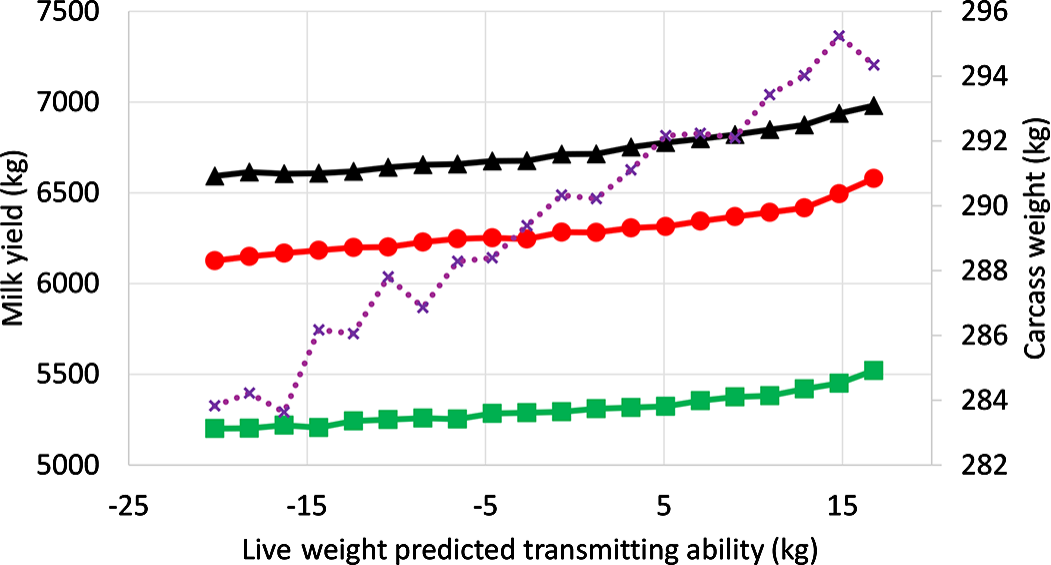
Mean 305-d milk yield for parity 1 (squares), parity 2 (circles), and parity 3 (triangles) cows of different live weight PTA; also included is the mean progeny carcass weight (broken line) from cows (across parities) differing in PTA; SE of the milk means were all ≤11.37 kg, whereas those for carcass weight were all ≤1.169 kg.

Although the association between cow live weight PTA stratum and milk solids yield differed (*P* < 0.001) by parity, the general trend of the association was relatively consistent by parity; the associations for parity 1 to 3 are in Figure 1 with the association for older parity animals following a similar pattern. Regardless of parity, there was a general tendency for milk solids yield to increase slightly with increasing genetic merit for live weight. The increase observed was a function of the increasing milk yield, but also of the reduction in milk fat concentration as live weight PTA increased (Figure 3); milk protein concentration, although associated (*P* < 0.001) with PTA stratum, did not vary much across live weight PTA strata. A 10-kg increase in PTA for live weight was associated with increases of 0.71%, 0.90%, and 0.81% in 305-d milk solids yield in parity 1, 2, and 3 cows, respectively. The SD of cow live weight used in the national genetic evaluations (divided by 2 to be on the PTA scale) is 13.2 kg. Also of note was that, within the range of live weight PTA explored in the present study, there was no obvious critical live weight below which milk solids yield declined at a faster rate; in fact, the strong coefficients of determination (R^2^ = 0.94, 0.93, and 0.95 for parities 1, 2, and 3, respectively) from linear regressions of milk solids yield on PTA stratum rank demonstrate that the relationship between milk solids yield and PTA for live weight was largely linear. This supports a predictable, proportional scaling of milk solids yield with live weight PTA. Accepting that a 1-kg difference in cow PTA is expected to translate to a 2-kg difference in phenotype (1.68 to 2.34 in the present study), a 100-kg genetically lighter cow is expected to, on average, yield 3.55% to 4.50% less milk solids yield per lactation. Importantly, though, when the predicted marginal means for phenotypic live weight and milk solids yield are considered together across the different live weight PTA strata, it is evident that the efficiency of milk solids production relative to cow live weight improves as cows become genetically lighter. The residual SD for milk solids did not vary considerably across PTA strata; the residual SD varied from 34.5 kg to 36.2 kg, except for the heaviest 2 strata, which were 37.5 kg and 39.2 kg.

**Figure 3:**
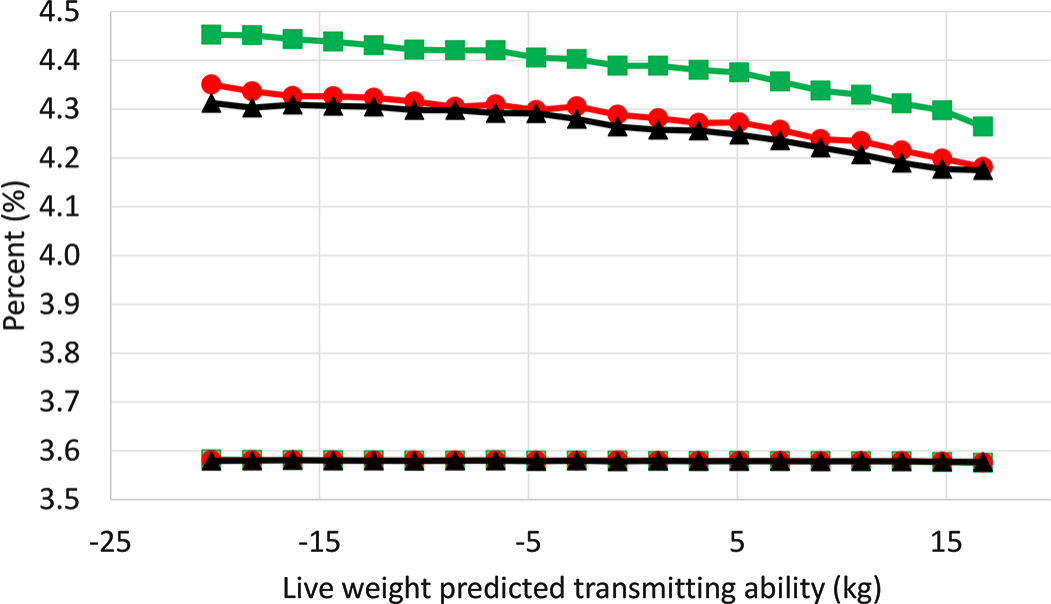
Mean 305-d fat percentage (top lines) and protein percentage (bottom lines) for parity 1 (squares), parity 2 (circles), and parity 3 (triangles) cows of different live weight PTA; SEM were all ≤0.0057.

The regression of progeny carcass weight on sire PTA for carcass weight was 0.84 (SE = 0.016), which is close to the expectation of 1. Although stratum of genetic merit for live weight was associated with progeny carcass weight, the association did not differ by parity (*P* = 0.34). The mean carcass weight of progeny increased relatively consistently as the cow (i.e., dam) got genetically heavier (Figure 2). This reflects a genetic correlation between cow size and progeny growth potential, likely arising from shared or coinherited alleles influencing growth, frame size, or muscularity. The association between cow genetic merit for live weight and progeny phenotypic carcass weight also suggests that in order to mitigate a reduction in progeny carcass weight born to genetically lighter dams, sires that produce heavier progeny carcasses (e.g., beef sires) should be used; the phenotypic difference in mean progeny carcass weight between cows at the extremes of genetic merit for live weight was 11.6 kg. The residual variance of carcass weight did not vary much by PTA strata with the residual SD being between 22.1 kg and 24.1 kg across the 20 live weight PTA strata.

This study was motivated by concerns that genetically lighter cows might produce less milk and have progeny with lighter carcasses. Also of interest was whether these associations followed a nonlinear pattern, where output might reduce more rapidly below a certain cow live weight threshold. Although genetically lighter cows did, on average, produce less milk (solids), the observed model effects were generally linear except at the very high live weight PTA, where there was a noticeable increase in output. Clearly, based on the data used in the present study from pasture-based Holstein-Friesian cows and the range in cow live weight PTA explored, there was no evidence of a critical live weight PTA threshold below which milk production reduced disproportionately. The difference in live weight PTA between the means of the extreme live weight PTA strata in the present study was 36.9 kg, which represents a difference of 2.80 genetic SD at the population level; assuming a mean of the population of zero, this represents a comparison of approximately the lightest 8.1% of possible individuals on genetic merit versus the heaviest 8.1% of individuals. In conclusion, given that the increase in output associated with genetically heavier cows was modest compared with the potential increased maintenance costs of heavier Holstein-Friesian cows, the economic advantage of selecting for heavier cows in pasture-based systems may be limited. Whether the study conclusions hold true either for other breeds or indeed other production systems warrants investigation.
